# Preclinical activity of SHR-A1921, a novel antibody-drug conjugate targeting trophoblast cell-surface antigen2 (Trop-2) in prostate cancer

**DOI:** 10.3389/fphar.2026.1713983

**Published:** 2026-03-03

**Authors:** Binyu Wang, Qiuya Xu, Shan Peng, Zheng Han, Haifeng Huang, Hongqian Guo, Xuefeng Qiu

**Affiliations:** 1 Department of Urology, Nanjing Drum Tower Hospital, Affiliated Hospital of Medical School, Nanjing University, Nanjing, Jiangsu, China; 2 Institute of Urology, Nanjing University, Nanjing, Jiangsu, China; 3 Department of Urology, Nanjing Drum Tower Hospital Clinical College of Nanjing University of Chinese Medicine, Nanjing, Jiangsu, China; 4 Department of Pathology, Nanjing Drum Tower Hospital, Affiliated Hospital of Medical School, Nanjing University, Nanjing, Jiangsu, China

**Keywords:** antibody-drug conjugates, patient-derived organoids, prostate cancer, SHR-A1921, targeted therapy, Trop-2

## Abstract

**Background:**

Despite advances in the 5-year survival rates for prostate cancer patients, progression to metastatic castration-resistant prostate cancer (mCRPC) remains a significant challenge following standard treatments. Antibody-drug conjugates (ADCs) are an emerging class of biopharmaceuticals that combine the specificity of monoclonal antibodies with the potency of cytotoxic drugs. Trophoblast cell surface antigen 2 (Trop-2) is overexpressed in prostate cancer, particularly in metastatic forms.

**Methods:**

Response to this, we developed a novel Trop-2-targeted antibody-drug conjugate (ADC), SHR-A1921, which incorporates a potent DNA topoisomerase I inhibitor,SHR9265. Its *in vitro* cytotoxicity was assessed across prostate cancer cell lines with differential Trop-2 expression. Subcutaneous xenograft models were established for *in vivo* tumor-suppressive activity evaluation, and patient-derived organoid models validated its potential clinical efficacy.

**Results:**

In preclinical models, SHR-A1921 specifically bound to Trop-2, followed by internalization into tumor cells and subsequent intracellular trafficking to lysosomes, where the release of SHR9265 occurred. This resulted in DNA damage and apoptosis in Trop-2-expressing tumor cells *in vitro*. *In vivo*, SHR-A1921 exhibited significant antitumor activity, inducing DNA damage in Trop-2-positive xenograft tumors. Additionally, SHR-A1921 demonstrated antitumor effects in Trop-2-expressing prostate cancer organoids. Safety assessments in rats indicated that SHR-A1921 had an acceptable safety profile.

**Conclusion:**

SHR-A1921 is a promising Trop-2-targeted ADC that leverages innovative technology to deliver potent antitumor activity against Trop-2-expressing prostate cancer cells, with an acceptable safety profile observed in preclinical studies. These results highlight the promising clinical potential of SHR-A1921 as a therapeutic option for prostate cancer patients with Trop-2-positive tumors.

## 1 Introduction

Prostate cancer is the most frequently diagnosed cancer in men worldwide and remains a major contributor to cancer-related deaths (Siegel et al., 2020). The standard treatment modalities for localized prostate cancer encompass surgical intervention, radiation therapy, and active surveillance (Sandhu et al., 2021; Sekhoacha et al., 2022; Fontana et al., 2020). Notably, the 5-year survival rate for localized disease is exceptionally high, reaching 98%, in stark contrast to the significantly lower rate of 30% observed in patients with metastatic disease (Desai et al., 2021). Addressing metastatic prostate cancer, patients typically undergo medical castration through frontline androgen deprivation therapy (ADT) utilizing agents like GnRH agonists or antagonists, often in combination with docetaxel or androgen pathway inhibitors such as abiraterone or enzalutamide (Scher et al., 2008; Teo et al., 2019). However, despite these treatments, a significant number of patients ultimately develop metastatic castration-resistant prostate cancer (mCRPC) (Teo et al., 2019; Calderon-Aparicio and Wang, 2021; Francini et al., 2025), leaving them with limited effective therapeutic options. It is evident that there exists a substantial unmet need for the development of novel and efficacious treatments for mCRPC.

In recent years, advancements in cancer treatment have introduced immunotherapy and poly (ADP-ribose) polymerase (PARP) inhibitors as effective options for specific subsets of prostate cancer patients (Mitsogiannis et al., 2022; Lakshmanan et al., 2020). Among the novel therapeutic approaches, Antibody-Drug Conjugates (ADCs) emerge as a highly promising treatment modality. ADCs consist of cytotoxic agents linked to antibodies that target antigens expressed on the surface of cancer cells, thereby minimizing exposure to healthy tissue and expanding the therapeutic window (Chau et al., 2019; Grairi and Le Borgne, 2024; Nicolò et al., 2022). Topoisomerase I (TOP1) inhibitors are widely utilized as cytotoxic payloads in ADCs. Topoisomerases, notably TOP1, facilitate DNA replication and transcription by modulating DNA topological states, and they serve as well-validated targets for antineoplastic therapy. Specifically, TOP1 inhibitors such as SHR9265 intercalate at the DNA-TOP1 interface, thereby abrogating the resolution of TOP1-induced single-strand breaks. This stabilization of the TOP1 cleavage complex (TOP1CC) subsequently triggers double-strand DNA breaks and ultimately leads to tumor cell death. Rapidly proliferating cells, particularly malignant counterparts, display heightened susceptibility to TOP1 inhibitors (Thomas and Pommier, 2019; Pommier and Thomas, 2023). Clinically, SHR9265 has been successfully integrated into ADCs such as SHR-A1811, a HER2-targeted ADC approved for the treatment of HER2-mutant non-small cell lung cancer (NSCLC) in 2025 (Li et al., 2025; Ma et al., 2025). As of July 2025, a total of 19 antibody-drug conjugates (ADCs) have received regulatory approval globally, targeting both hematological malignancies and solid tumors. Furthermore, more than 100 ADC candidates are presently undergoing evaluation across different stages of clinical trials (Fu et al., 2022). The advent of ADC drugs marks a new era in targeted cancer therapy, offering hope for improved outcomes and reduced side effects.

Trop-2, a multifunctional glycoprotein encoded by the TACSTD2 (tumor-associated calcium signal transducer 2) gene, is involved in a range of physiological and pathological processes (Qiu et al., 2023). Studies indicate that Trop-2 expression is consistently elevated in various forms of cancer, independent of its expression levels in normal cells (Trerotola et al., 2013a). The increased expression of Trop-2 has been linked to enhanced tumor growth and is associated with a worse prognosis (Koltai and Fliegel, 2023). The broad expression of Trop-2 has prompted significant interest in its potential as a therapeutic target for various cancers (Liu et al., 2024; Morgenstern-Kaplan et al., 2024). Sacituzumab govitecan (SG) was the first FDA-approved therapy, with other agents, such as datopotamab deruxtecan (Dato-DXd), currently undergoing clinical evaluation (Qiu et al., 2023). Preclinical studies have demonstrated that Trop-2 is significantly upregulated in both primary and metastatic prostate cancer tissues compared to benign luminal cells (Shen et al., 2021). Trop-2 regulates the β1 integrin-mediated adhesion of prostate cancer cells to fibronectin, acting as an anti-adhesive molecule on this extracellular matrix ligand. This implies that Trop-2 may contribute to the metastatic progression of prostate cancer cells (Trerotola et al., 2013b). A recent study found no significant change in TACSTD2 expression in patients who had previously been treated with androgen receptor signaling inhibitors (ARSI). However, Trop-2 has been identified as a potential cell surface target for isolating circulating tumor cells (CTCs). This suggests that therapies targeting Trop-2 may hold promise for effectively treating men with mCRPC or metastatic disease (Sperger et al., 2023).

In this report, we demonstrated that preclinical activity of SHR-A1921, a humanized ADC targeting Trop-2. SHR-A1921 exhibited cytotoxicity toward Trop-2 positive human prostate cell lines and antitumor activity in preclinical tumor models and organoid.

## 2 Materials and methods

### 2.1 Drug information

**FIGURE 1:**
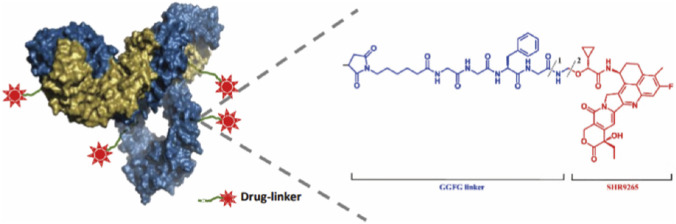
The detail information about SHR-A1921.

SHR-A1921 was synthesized by SHR-1920 and SHR169106. SHR-1920 is humanized Trop-2 IgG1 monoclonal antibody. SHR169106 is composed of an enzyme-cut linker containing maleimide tetrapeptide (GGFG) with DNA topoisomerase I inhibitor (SHR9265). SHR-A1921 is a mixture of antibody conjugations and the target drug antibody conjugations ratio (DAR) is 4 (Figure 1).

### 2.2 Cells

The cell lines PC3, 22RV1, DU145, LNCaP, and C4-2, all of which were obtained from ATCC, were cultured in RPMI1640 medium supplemented with 10% fetal bovine serum (FBS). The cells were maintained at 37 °C in a 5% CO2 atmosphere.

### 2.3 Hematoxylin-eosin (H&E) and immunohistochemical (IHC) analysis

IHC analysis of paraffin-embedded tissue specimens was carried out in accordance with the manufacturer’s previously specified experimental protocols (Jiang et al., 2022). Images were captured with a NanoZoomer S60 slide scanner (Hamamatsu). All antibodies used were listed in Supplementary Table S2. The mean optical density (MOD) of Trop-2 expression was quantified using QuPath software (Version 0.6.0).

### 2.4 Western blotting

Protein extraction and Western blot were performed following our previously published protocol (Yin et al., 2022). Antibodies used were presented in Supplementary Table S2. Protein detection was performed using an electrochemiluminescence (ECL) system (Tanon Co., Ltd.). Protein band intensities were quantified with ImageJ software to calculate the gray values.

### 2.5 Flow cytometry

Cells were trypsinized briefly, washed, and resuspended in 1% BSA-PBS for Trop-2 expression analysis. All antibodies used in the assays are listed in Supplementary Table S2. All incubations were carried out for 45 min at 4 °C, with 1% BSA-PBS washes between each incubation step. Flow cytometry was performed using a BD FACSCalibur (BD Biosciences, San Jose, CA) to assess cell binding.

### 2.6 *In vitro* cellular toxicity assays

Cells were trypsinized, plated in 96-well plates at 2,000 cells per well, and incubated overnight at 37 °C. After 24 h, the cells were exposed to the designated compounds for 6 days and cell viability was assessed via the MTT assay.

### 2.7 *In Vivo* therapeutic studies

Cell line-derived xenograft (CDX) models were generated in male athymic nude mice (nu/nu), 5 weeks old, obtained from GemPharmatech. Mice were randomly allocated to the control or treatment group, with treatment commencing on day 0 once tumor volume reached approximately 100 mm^3^. SHR-A1921, SHR-1920, or PBS was administered intraperitoneally on day 0. Direct administration of SHR9265 to mice was omitted, as its potent systemic and non-specific cytotoxicity would confound the *in vivo* evaluation. Tumor volume, determined using the formula 1/2 × length × width^2^, was assessed three times weekly. The tumor sizes were then compared between the control and treatment groups to evaluate the therapeutic efficacy of the treatment.

### 2.8 Organoid culture

Tissue samples were transferred to 50 mL centrifuge tubes and washed 3–5 times with 20–30 mL sterile saline containing 1% diamantine and 50 µg/mL gentamicin. The tissues were minced into 1 mm pieces using sterile scissors on ice and collected in a 15 mL centrifuge tube with HBSS. Following centrifugation, the supernatant was removed, 3 mL of digestive solution was added and incubated at 37 °C in a shaking incubator for 0.5–3 h to facilitate digestion. Digestion was stopped by adding 5 mL HBSS, followed by centrifugation and removal of the supernatant. The tissue was further digested with 2 mL preheated digestive fluid II for 5–10 min at 37 °C. Digestion was stopped by adding HBSS, followed by centrifugation. The cell pellet was resuspended in 5 mL HBSS, filtered through a 100 µm strainer, then centrifuged to collect cells. The pellet was incubated with 2 mL erythrocyte lysate for 2–5 min, then diluted with 4 mL HBSS to terminate dissociation. The cells were centrifuged, and a suspension of 10^4–10^5 cells/30 µL was prepared in culture medium. An appropriate volume (no more than 200 µL) of pre-melted collagen P and cell suspension (1:1.5 ratio) was mixed thoroughly, then pipetted into a 60 mm culture dish in 50 µL drops. The dish was incubated at 37 °C for 2 min, inverted after the mixture solidified (approx. 30 min), and cultured in a CO2 incubator at 37 °C. Patient-derived organoids (PDOs) were typically obtained in 4–10 days.

### 2.9 Organoid drug

Prostate cancer organoids were harvested and dissociated using TrypLe (Gibco, United States). Dissociated organoids were combined with Matrigel (Corning) in OCOM (Accuroid, China) (1:1, v/v) and added to 384-well black plates with clear bottoms (Corning, United States). After gelation, 50 µL organoid culture media was added to each well. The organoids were treated with SHR-A1921 for 6 days. Cell viability was assessed using 3D CellTiter-Glo (Promega, United States).

### 2.10 Statistical analysis

Statistical analyses were carried out with GraphPad Prism 9 software (GraphPad Software, Inc., San Diego, California, United States). All quantitative data were generated from a minimum of three independent experimental replicates, and presented as the mean ± standard deviation (SD). Comparisons of continuous variables were conducted via the Student’s t-test, where statistical significance was defined as *p < 0.05; **p < 0.01; ***p < 0.001; ****p < 0.0001.

## 3 Results

### 3.1 Trop-2 expression on human prostate cancer

**FIGURE 2:**
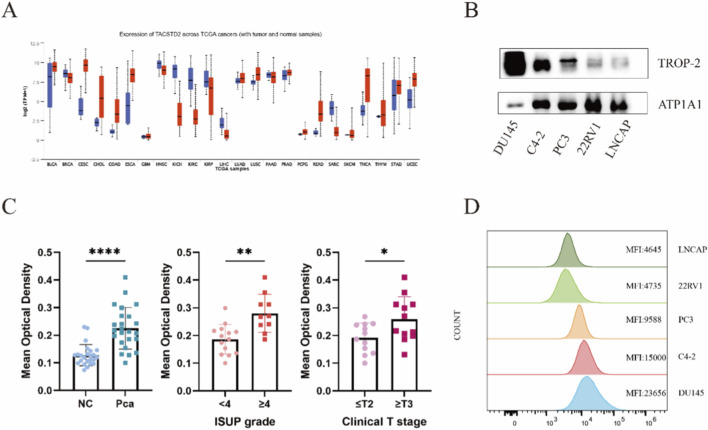
TACSTD2 RNA and protein expression in Prostate carcinomas. **(A)** Median TACSTD2 expression in TCGA RNA-seq by cancer type. **(B)** Western blot of Trop-2 protein expression in the membrane of a panel of prostate cancer cell lines of different molecular subtypes. **(C)** Trop-2 expression profiling in normal prostate tissues and prostate carcinomas with different ISUP grades and clinical T stages identified by TMA. Quantitative analysis of TROP-2 expression levels assessed by mean optical density (MOD). Data are presented as mean ± standard deviation (SD). Statistical significance was determined using unpaired t-test. (*P < 0.05, **P < 0.01, ***P < 0.001, ****P < 0.0001). **(D)** Flow cytometry of Trop-2 protein expression in the membrane of a panel of prostate cancer cell lines of different molecular subtypes.

The expression of TACSTD2 across TCGA cancers (including tumor and normal samples) was shown in Figure 2A, Trop-2 expression upregulate in tumor sample. A tissue microarray (TMA) containing 24 pairs of prostate cancer (PCa) tissues and their adjacent non-tumor counterparts was constructed in this study. The Gleason scores of the PCa tissues ranged from 3 + 3 to 4 + 5 (Supplementary Figure S1). Hematoxylin-eosin (HE) staining and immunohistochemical (IHC) staining were performed on the TMA (Supplementary Figure S1), and the mean optical density (MOD) of Trop-2 expression was quantified for each sample. Statistical analysis revealed that the MOD of Trop-2 in PCa tissues was significantly higher than that in adjacent non-tumor tissues (P < 0.0001). Further subgroup analysis of the 24 PCa specimens demonstrated that Trop-2 expression was notably elevated in tissues with an ISUP grade ≥4 (P < 0.001) and advanced clinical T stage (P < 0.05) (Figure 2C).

Flow cytometry and Western blotting were used to assess the cell surface expression of Trop-2 across several human prostate cancer cell lines (Figures 2B,D). A mean fluorescence intensity (MFI) greater than 20,000 was considered indicative of 3+ Trop-2 expression, those with 10,000–20,000 were 2+, those with 5,000–10,000 were 1+, and those with 5,000 or less were 0. DU145 was found to show 3+, whereas C4-2 showed 2++, PC3 showed 1+, and 22RV1 and LNCaP showed 0 (Figure 2D).

### 3.2 *In vitro* antitumor activity of SHR-A1921

SHR-A1921 is an antibody-drug conjugate (ADC) consisting of a recombinant humanized anti-Trop-2 IgG1 monoclonal antibody conjugated with a DNA topoisomerase I inhibitor (SHR9265) via a GGFG linker. Upon internalization, this linker is cleaved by lysosomal cathepsin enzymes. Surface plasmon resonance (SPR) technology was employed to determine the binding affinities of SHR-A1921 to Trop-2 proteins from different species, including humans, rhesus monkeys, rats, and mice. As shown in Table 1, the results demonstrated that SHR-A1921 binds specifically to human and rhesus monkey Trop-2 with comparable high affinities at the nanomolar level (10^–10^ M), while no binding was detected with rat or mouse Trop-2. Consistent with this species selectivity, SHR-1920 also exhibited binding specificity exclusively for human and rhesus monkey Trop-2, with an affinity profile similar to that of SHR-A1921.

**TABLE 1:** Evaluation of the binding ability of SHR-A1921 to Trop-2 from different species.

Drug name	Antibody	Ka (1/Ms)	Kd (1/s)	K_D_ (M)
SHR-A1921 SHR-1920	Human Trop-2	3.01E+05 3.22E+05	2.13E-04 2.04E-04	7.09E-10 6.34E-10
SHR-A1921 SHR-1920	Rhesus macaque Trop-2	4.49E+05 4.62E+05	2.26E-04 2.27E-04	5.04E-10 4.98E10
SHR-A1921 SHR-1920	Rat Trop-2	-	-	-
SHR-A1921 SHR-1920	Mouse Trop-2	-	-	-

**FIGURE 3:**
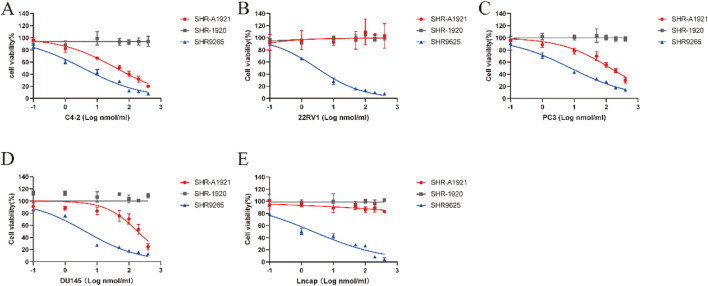
SHR-A1921 specifically target and kills Trop-2 expressing cells *in vitro*. **(A–E)** The cytotoxicity of SHR-A1921, SHR-1920 and SHR9265 in a cell viability assay. Cell were treated with 0.1–400 nmol/L of indicated drug and all viability were normalized to vehicle. Date represented as mean ± SEM for three independents, three technical replicates per experiment.

*In vitro* antitumor testing involved analyzing growth inhibition activity after 6 days of treatment with increasing concentrations of SHR-A1921, SHR-1920, and SHR9265 on Trop-2-positive prostate cancer cell lines (C4-2, DU145, PC3) and Trop-2 negative prostate cancer cell lines (22RV1 and Lncap) (Figures 3A–E). Trop-2-positive cell lines exhibited sensitivity to SHR-A1921, while Trop-2-negative cell lines demonstrated comparative resistance. This dose-dependent correlation between Trop-2 expression and drug sensitivity strongly supports that the antitumor effect is mediated by Trop-2. Notably, the half-maximal inhibitory concentration (IC_50_) of SHR-A1921 against C4-2 cells (29.75 ± 0.85 nM) was unexpectedly lower than that against DU145 cells (177.75 ± 0.85 nM), despite the higher Trop-2 expression level in DU145 cells. This finding suggests that Trop-2 expression is not the sole determinant of the therapeutic efficacy of SHR-A1921 (Table 2). We further analyzed the correlation between the antitumor efficacy of SHR-A1921 at a concentration of 400 nM and the cellular Trop-2 expression level (assessed by MFI). Our findings indicate that a Trop-2 expression threshold of ≥8,300 is required for SHR-A1921 to exert optimal antitumor activity at the 400 nM concentration (Supplementary Figure S2).

**TABLE 2:** *In vitro* cell growth inhibitory activity of SHR-A1921.

Cell name	IC50 (nM)		
	SHR-A1921	SHR-1920	SHR9265
C4-2	29.75 ± 0.85	>400	3.71 ± 0.96
DU145	177.75 ± 2.05	>400	4.29 ± 0.36
PC3	137.53 ± 26.06	>400	7.74 ± 1.99
22RV1	>400	>400	3.04 ± 0.47
Lncap	>400	>400	2.41 ± 0.59

### 3.3 Antitumor activity of SHR-A1921 in cell line-derived xenograft models

**FIGURE 4:**
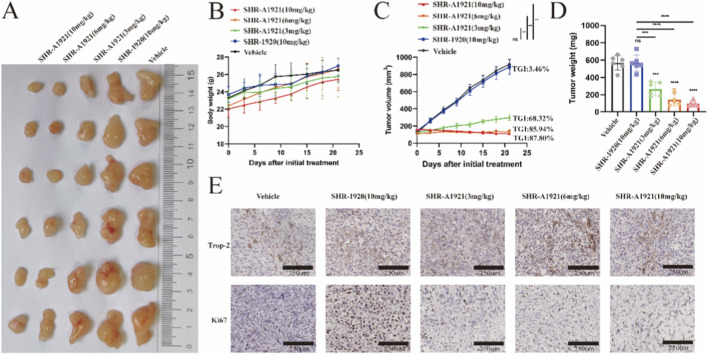
Trop-2 ADC induce antitumor activity in Trop-2 expression prostate cancer lines derived xenograft models. **(A–D)** PC3 tumor cells were inoculated into nude mice, and the mice were treated with SHR-A1921 (3 mg/kg, 6 mg/kg, 10 mg/kg), SHR-1920 (10 mg/kg) and Vehicle. Tumor growth and body weight were measured. **(E)** IHC staining of tumor tissue with the Abs of Ki67(proliferation) demonstrated an decrease in tumor cell proliferation treated with SHR-A1921 (3 mg/kg, 6 mg/kg, 10 mg/kg), SHR-1920 (10 mg/kg) and Vehicle.

*In vitro* studies revealed that C4-2, PC3, and DU145 cells exhibit sensitivity to SHR-A1921 compared to 22RV1 cells. Subsequently, a prostate tumor model was established in immunodeficient nude mice using these cells to assess efficacy *in vivo*. In a preliminary tolerability experiment, mice with PC3 tumors of approximately 3.5 mm in diameter were treated with 3, 6, and 10 mg/kg SHR-A1921 or vehicle (PBS). No signs of distress or weight loss were observed in mice treated with SHR-A1921, indicating absence of dose-limiting toxicities (Figure 4B; Supplementary Figure S8). A dose-dependent inhibition of tumor growth was noted in mice treated with SHR-A1921, with almost complete tumor regression observed at 10 mg/kg dose and 6 mg/kg dose (Figures 4A-D). Tumor staining at necropsy confirmed retention of trop-2 expression *in vivo*, suggesting continued sensitivity to further ADC treatment (Figure 4E).

**FIGURE 5:**
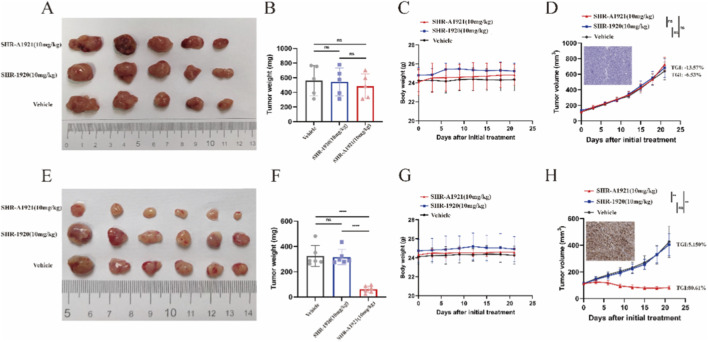
Trop-2 ADC potently and selectively kills Trop-2 high prostate cancer cell lines derived xenograft models. **(A–D)** 22RV1 tumor cells were inoculated into nude mice, and the mice were treated with SHR-A1921, SHR-1920 and Vehicle. Tumor growth and body weight were measured. **(E–H)** DU145 tumor cells were inoculated into nude mice, and the mice were treated with SHR-A1921, SHR-1920 and Vehicle. Tumor growth and body weight were measured.

Based on these findings, nude mice were subcutaneously inoculated with C4-2, DU145, and 22RV1 cells. Mice were intravenously administered vehicle (PBS), SHR-1920, and SHR-A1921 (all at 10 mg/kg). No toxicity was observed with any treatment, as indicated by monitoring of animal body weight and clinical signs (Figures 5C,G; Supplementary Figures S5–S7). Treatment with SHR-A1921 (TGI = −13.57%) or SHR-1920 (TGI = −6.53%) did not affect 22RV1 tumor growth rate, final tumor size, or weight compared to vehicle-treated mice (Figures 5A–D). However, SHR-A1921 treatment resulted in tumor regression in C4-2 or DU145 tumors compared to SHR-1920 and vehicle-treated mice (Figures 5E–H; Supplementary Figures S3A–D). Immunohistochemical analysis confirmed primary tumor regression from SHR-A1921-treated C4-2 and DU145 tumors (Supplementary Figure S4). Table 3 summarizes the tumor growth inhibition (TGI) rates of SHR-A1921 in three patient-derived xenograft (PDX) models. In summary, these findings demonstrate that SHR-A1921 effectively induces primary tumor regression in mice bearing C4-2 and DU145 tumors.

**TABLE 3:** Summary of tumor growth inhibition of SHR-A1921.

Tumor model	Trop-2 expression	Administration		Tumor growth inhibition (%)
		Dose (mg/kg)	Time (day)	
22RV1	+	10	21	−13.57
DU145	+++	10	21	80.16
C4-2	++	10	21	78.60

### 3.4 Drug responses of prostate cancer organoids

**FIGURE 6:**
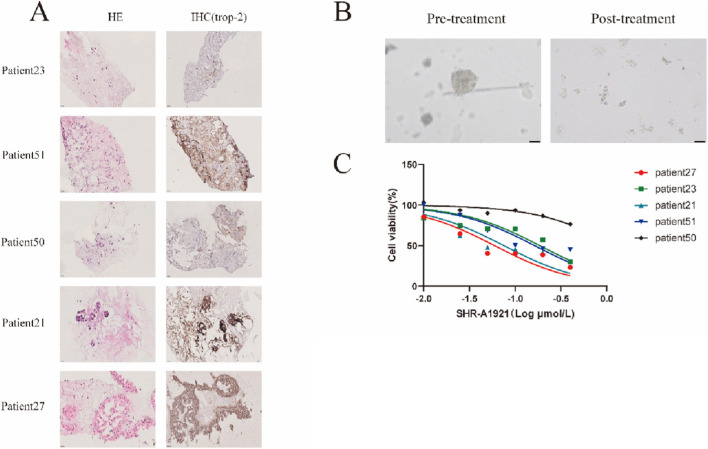
Trop-2 ADC induce antitumor activity in Trop-2-high patient-derived tumor organoids. **(A)** IHC of trop-2 expression in patient-derived tumor organoids (PDOs) of prostate carcinoma. **(B)** Confocal imaging showing PDOs after 6 days of treatment with Trop-2 ADC. **(C)** Cytotoxicity assay of PDOs treated with Trop-2 ADC.

Building on the findings from both *in vivo* and *in vitro* experiments, prostate cancer organoids were employed as advanced preclinical models to assess tumor responses to various anticancer agents. These organoids, which closely mimic the complexity and heterogeneity of human prostate cancer, were utilized to evaluate the therapeutic potential and efficacy of SHR-A1921. Drug screenings were performed on 5 prostate cancer organoid lines. Tumoroids were treated with a dilution series of SHR-A1921 for 6 days, before measuring cell viabilitty50. Drug sensitivity was represented by the half-maximal inhibitory concentration (IC_50_). Trop-2 was expressed on some patient-derived prostate cancer organoids verified by IHC (Figure 6A) while organoids with high expression of Trop-2 showed more sensitive to SHR-A1921 (Figure 6C). Confocal imaging showing PDOs after 6 days of treatment with Trop-2 ADC (Figure 6B), indicating the cytotoxicity of SHR-A1921 to organoids. The result indicates that some prostate cancer patients especially with high expression of Trop-2 can benefit from SHR-A1921 (Table 4).

**TABLE 4:** IC50 of SHR-A1921 and positive area of Top-2 in organoid in IHC.

​	Patient 27	Patient 23	Patient 21	Patient 51	Patient 50
IC50 (μmol/L)	0.060	0.213	0.080	0.184	1.949
Trop-2 (%Area)	22.515	2.103	16.266	7.656	0.823

## 4 Discussion

Antibody-drug conjugates (ADCs) that target tumor-specific antigens represent an emerging and promising class of targeted treatments, showing substantial effectiveness in various cancers. By linking monoclonal antibodies with potent chemotherapy agents, ADCs offer selective tumor cell targeting while reducing harm to healthy tissues (Parslow et al., 2016; Beck et al., 2017). Trop-2, also known as membrane component surface marker-1, holds promise as a target for ADC therapy due to its widespread expression in numerous cancer types and limited expression in normal tissues (Zaman et al., 2019; Guerra et al., 2023). However, only a limited number of therapeutic agents directly targeting Trop-2 have been approved to date (Tarantino et al., 2022; Fuentes-Antrás et al., 2023; Thomas et al., 2016).

In response to this gap, we engineered a humanized anti-Trop-2 antibody capable of specifically binding to Trop-2, internalizing post-antigen binding, and trafficking to the lysosome. Using this antibody, we developed and synthesized SHR-A1921, which harnesses the antibody’s specificity and the potency of a microtubule-disrupting agent to create a Trop-2-directed cytotoxic agent. Here, we demonstrate its efficacy in preclinical prostate cancer models, highlighting its potential as a promising therapeutic option for prostate cancer patients.

In both *in vitro* and *in vivo* studies, SHR-A1921 exhibited strong antitumor effects by inducing DNA damage and apoptosis specifically in prostate cancer models with high Trop-2 expression, while showing minimal effects in Trop-2 low models. The action mechanism of ADCs includes several critical steps such as target binding, antibody internalization, intracellular trafficking, and payload release (Hedrich et al., 2018; Khoury et al., 2023), all contributing to the therapeutic effectiveness. In line with this mechanism, SHR-A1921 was efficiently internalized into Trop-2 expressing prostate cancer cells and transported to lysosomes, resulting in the release of SHR9265. However, as potential unidentified factors may influence the molecular dynamics and antitumor activity of SHR-A1921, further research is required to determine the necessary Trop-2 expression level for effective SHR-A1921 activity. Moreover, more detailed comparisons with other Trop-2-targeted ADCs, such as Sacituzumab govitecan, are needed to assess efficacy and safety.

A drug-to-antibody ratio (DAR) of 4 was selected for SHR-A1921 as the optimal configuration to maximize its therapeutic window. However, the differential effects of SHR-A1921 on tumor cells versus normal cells are still being thoroughly investigated. Preliminary findings suggest that the overexpression of Trop-2 in tumor cells, combined with the tumor-selective tetrapeptide-based linker, may promote the targeted release of SHR9265 within cancer cells (Xi et al., 2024; Cheng-Sánchez et al., 2022). Additionally, the rapid proliferation of cancer cells likely increases their sensitivity to topoisomerase inhibitors, such as SHR9265, compared to slower-growing normal cells.

The efficacy of SHR-A1921 correlated with the Trop-2 expression level on prostate cancer cell surfaces, though there was variability among Trop-2 positive cell lines (C4-2, DU145, PC3). The observed variation in internalization and release of SHR9265 may be attributed to differences in intracellular trafficking mechanisms, including clathrin- or caveolin-mediated endocytosis, as well as variations in lysosomal enzyme activities (Ravi and McGregor, 2021). In addition to the effective delivery of SHR9265, the inherent sensitivity of tumor to this compound is another pivotal factor that determines the therapeutic potency of SHR-A1921 (García-Alonso et al., 2018). Notably, SHR9265 showed strong growth inhibitory activity in all prostate cancer cell lines, with IC50 values ranging from 2.41 to 7.74 nM.

While the primary objective of this study was to evaluate the efficacy of SHR-A1921 as a novel therapeutic approach for prostate cancer, several limitations should be acknowledged. First, the use of an ADC targeting human Trop-2 required the implantation of human tumors in immunocompromised mice, which restricts the evaluation of potential on-target off-tumor toxicities and immune responses. Although no adverse effects were observed in terms of body weight changes or signs of illness in SHR-A1921-treated mice, suggesting the ADC’s stability and lack of off-target toxicities, it is important to recognize that Trop-2 is also expressed in normal prostate tissue, albeit at lower levels compared to carcinoma. Therefore, further studies are needed to explore any possible on-target off-tumor toxicities. Second, the use of non-specific ADCs as controls limits the ability to assess whether the SHR9265 conjugated with IgG influences prostate tumor cells with high Trop-2 expression.

In summary, SHR-A1921 demonstrates selective targeting of Trop-2 expressing prostate cancer cell lines, resulting in tumor regression in both preclinical models and patient-derived organoids. These results underscore the potential of SHR-A1921 as a promising therapeutic option for prostate cancer patients with high Trop-2 expression.

## Data Availability

The raw data supporting the conclusions of this article will be made available by the authors, without undue reservation.
