# Supplementary material for: Preclinical activity of SHR-A1921, a novel antibody-drug conjugate targeting trophoblast cell-surface antigen2 (Trop-2) in prostate cancer
Source: Front Pharmacol. 2026 Mar 3;17:1713983. doi: 10.3389/fphar.2026.1713983 (PMC13040357; doi:10.3389/fphar.2026.1713983)

**Supplemental materials**

**Figure1 HE staining and IHC staining on the TMA.** TMA containing 24 pairs of prostate cancer (PCa) tissues and their adjacent non-tumor counterparts The Gleason scores of the PCa tissues ranged from 3+3 to 4+5.The mean optical density (MOD) of Trop-2 expression was quantified for each sample.

**Figure2 Association between Trop-2 expression levels in PCa cell lines and the responsiveness to SHR-A1921 at a concentration of 400 nM.** Correlation analysis between Trop-2 MFI values and relative cell viability after 400 nM SHR-A1921 treatment.

**Figure3** **Trop-2 ADC potently and selectively kills C4-2 derived xenograft models. (A-D)** C4-2 tumor cells were inoculated into nude mice, and the mice were treated with SHR-A1921, SHR-1920 and Vehicle. Tumor growth and body weight were measured.

**Figure4 Primary tumor regression from SHR-A1921-treated in IHC** Immunohistochemical staining (Ki67) of tumor in CDX mice were treated with SHR-A1921.

**Figure5-8 Safety Assessment of Trop-2 ADC in prostate cancer cell lines derived xenograft models.** Representative hematoxylin and eosin staining of organs (heart, liver, spleen, lung, kidney) in CDX mice were treated with SHR-A1921, SHR-1920 and Vehicle. (Figure5 represents 22RV1, Figure6 represents C4-2, Figure7 represents DU145, Figure8 represents PC3)

**Supplemental Figure**

**Supplemental Figure1**


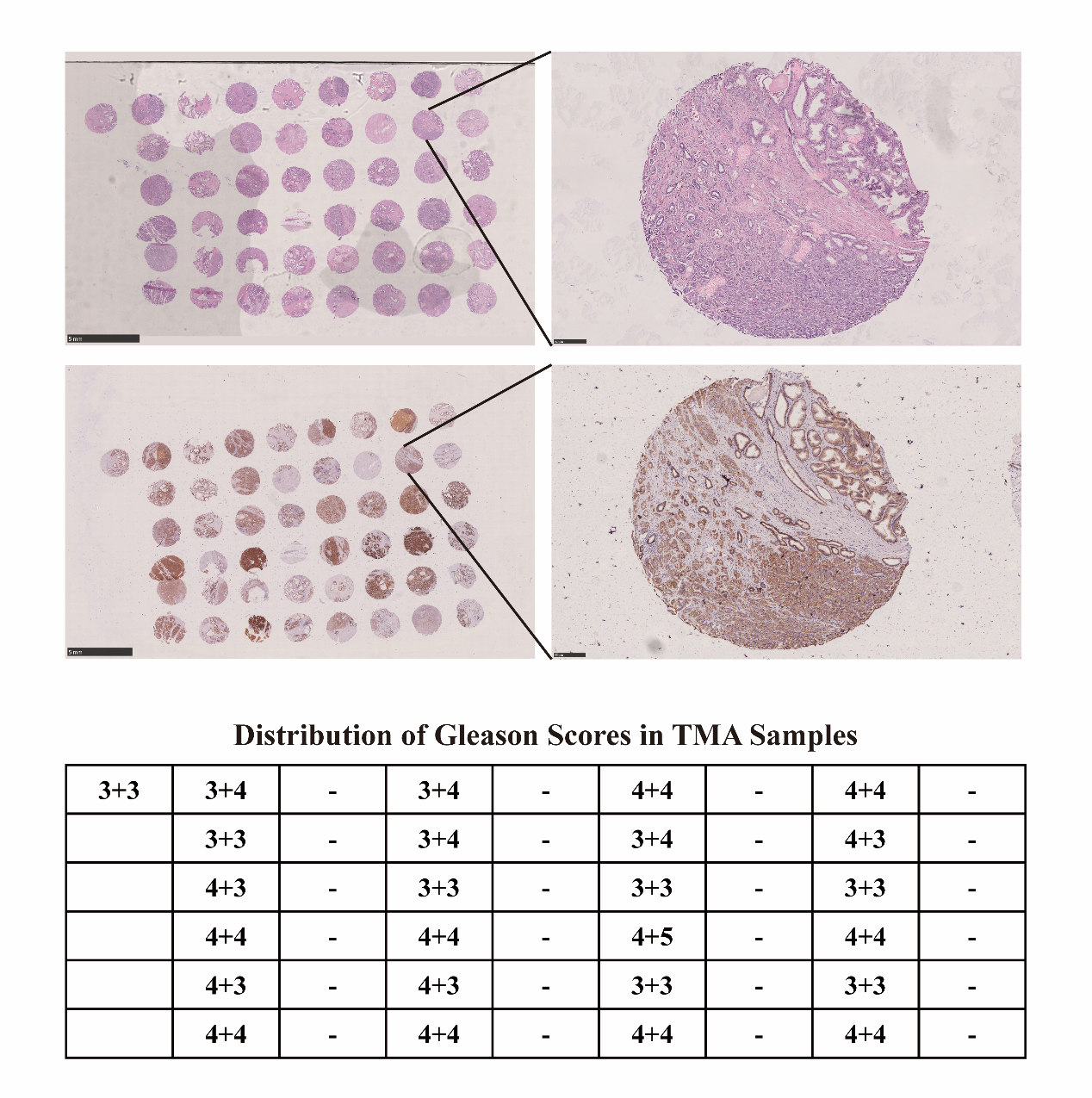


**Supplemental Figure2**

**
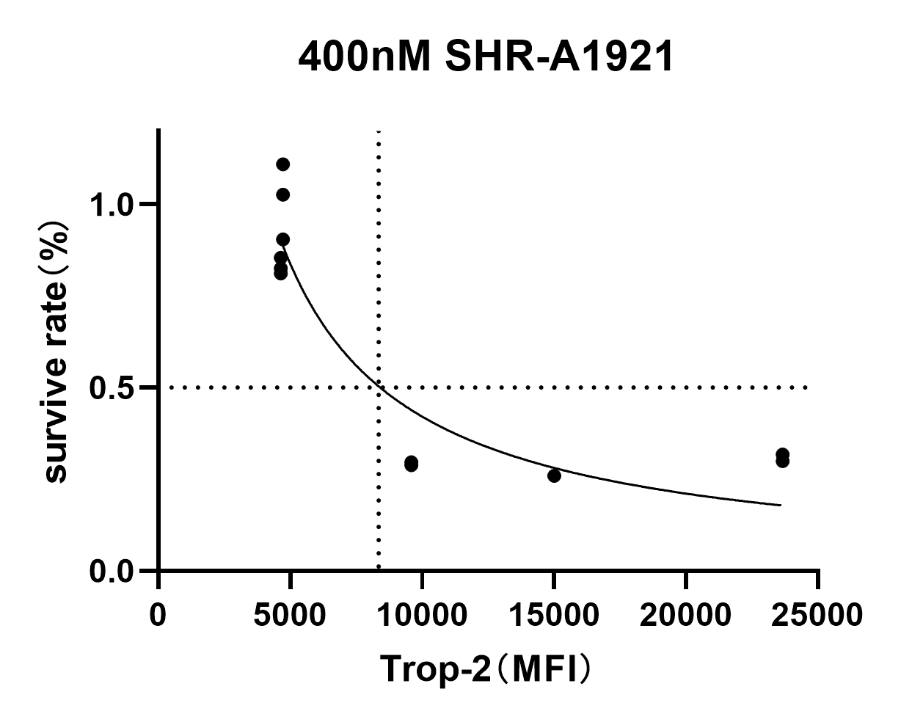
**

**Supplemental Figure3**


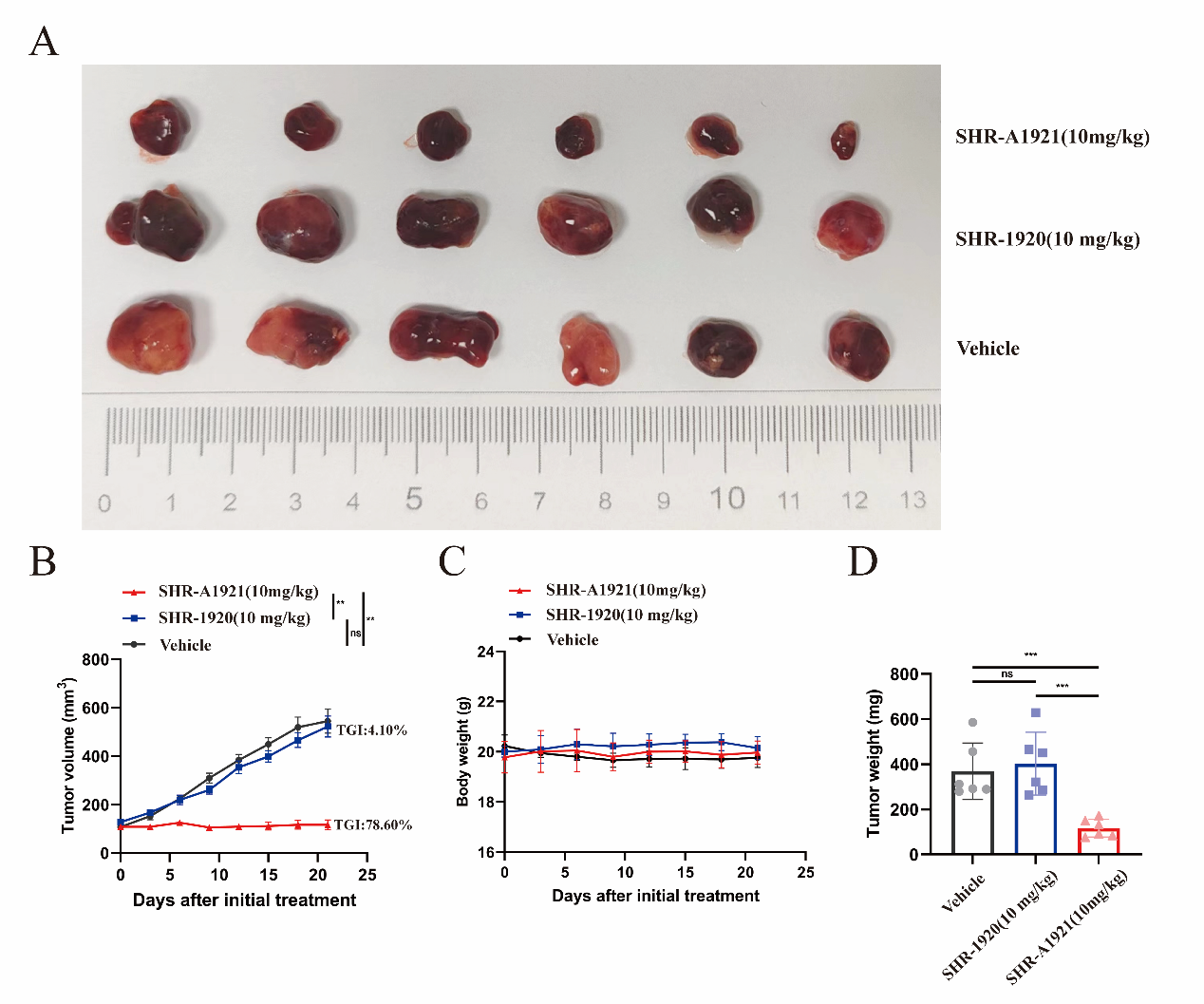


**Supplemental Figure4**


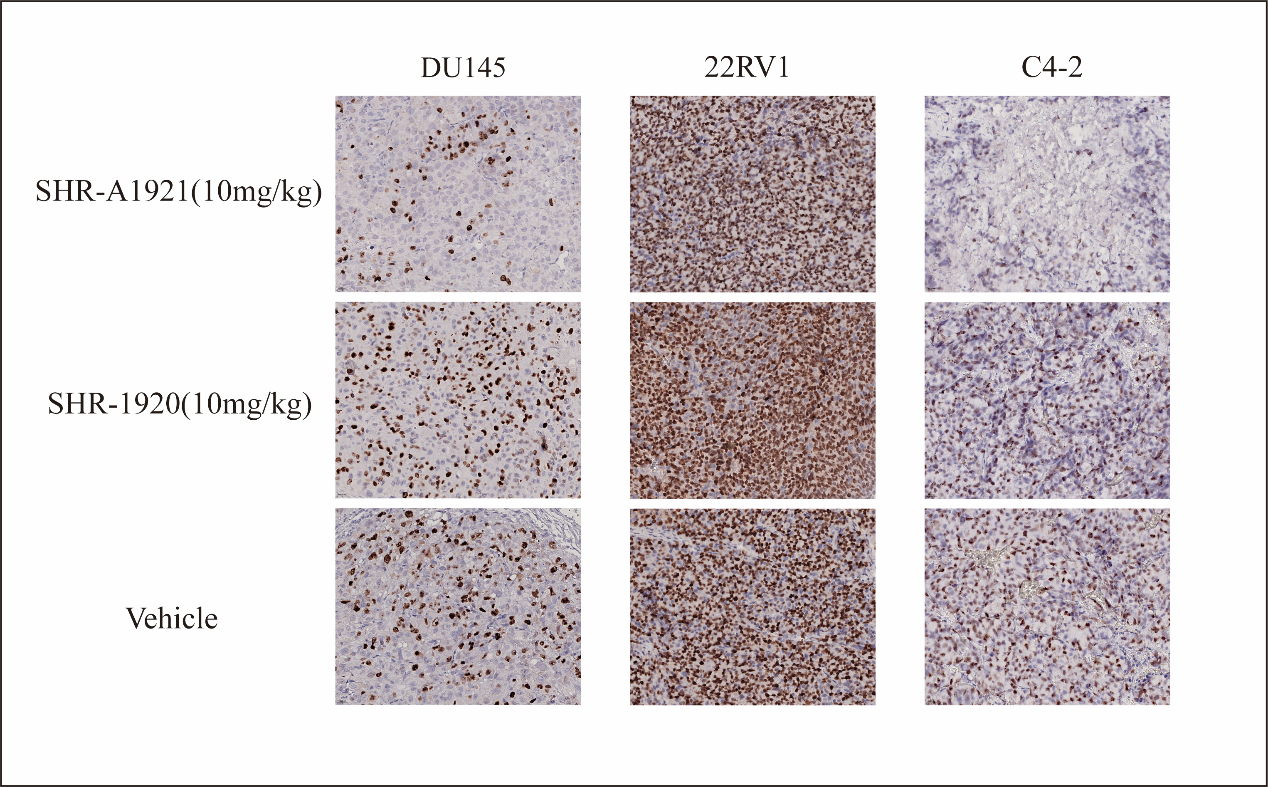


**Supplemental Figure5**

**
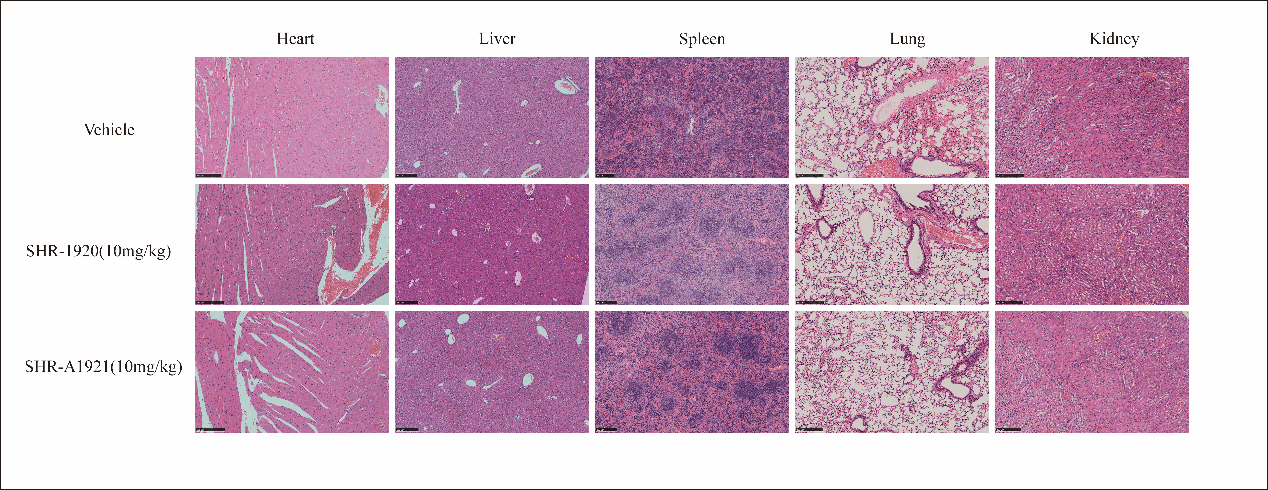
**

**Supplemental Figure6**

**
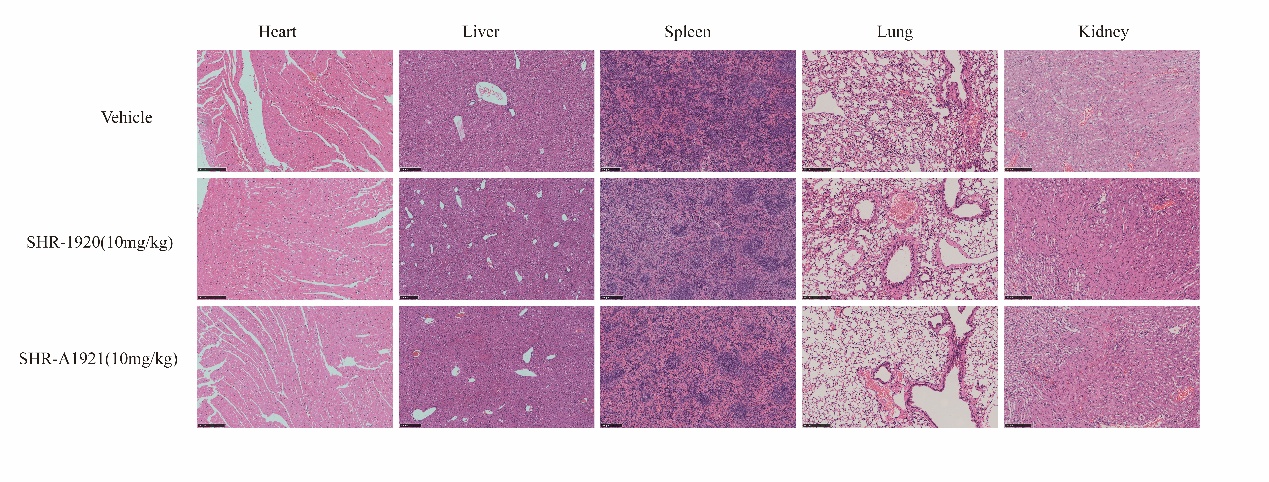
**

**Supplemental Figure7**

**
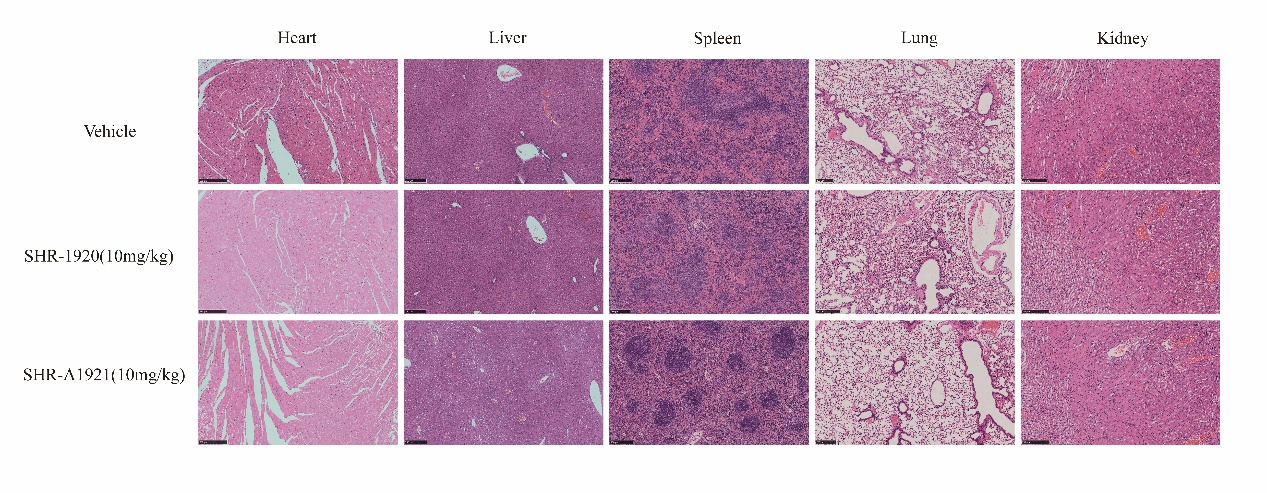
**

**Supplemental Figure8**


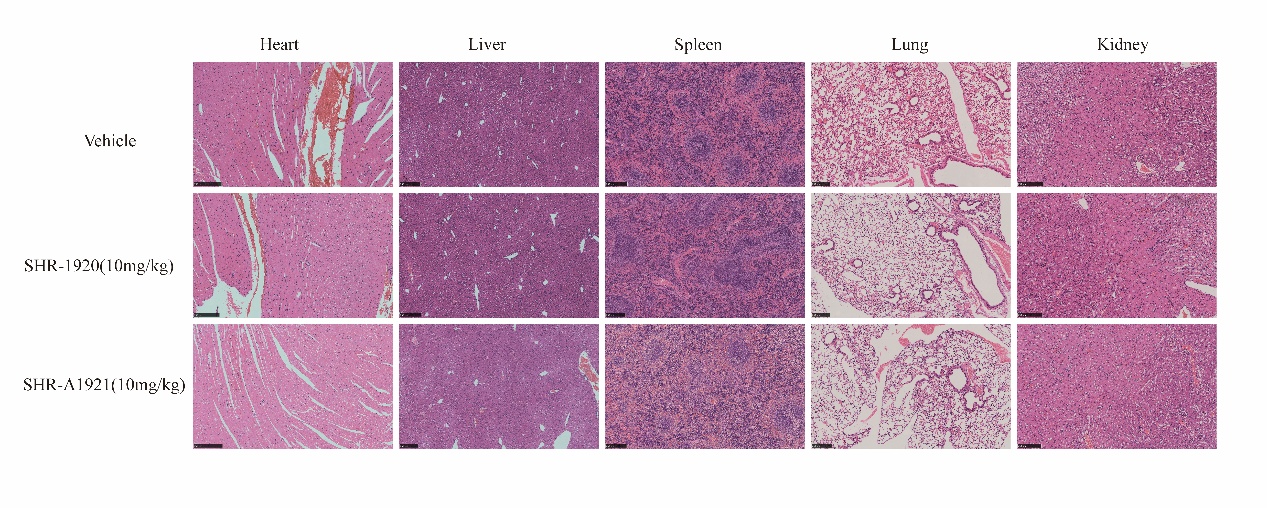


**Supplemental Table1**


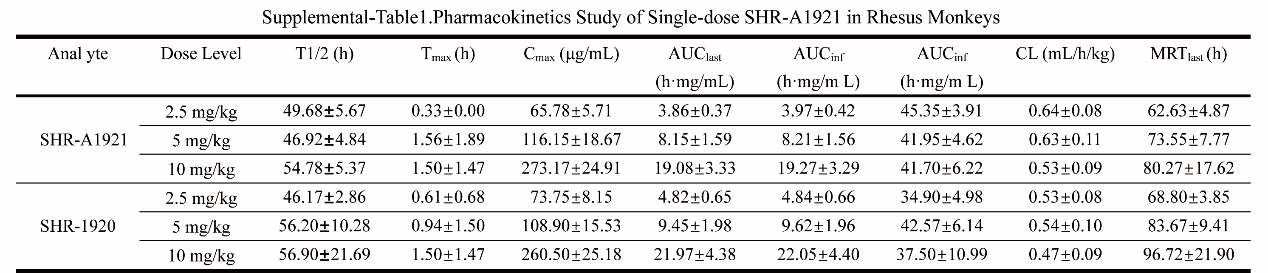


**Supplemental Table2**


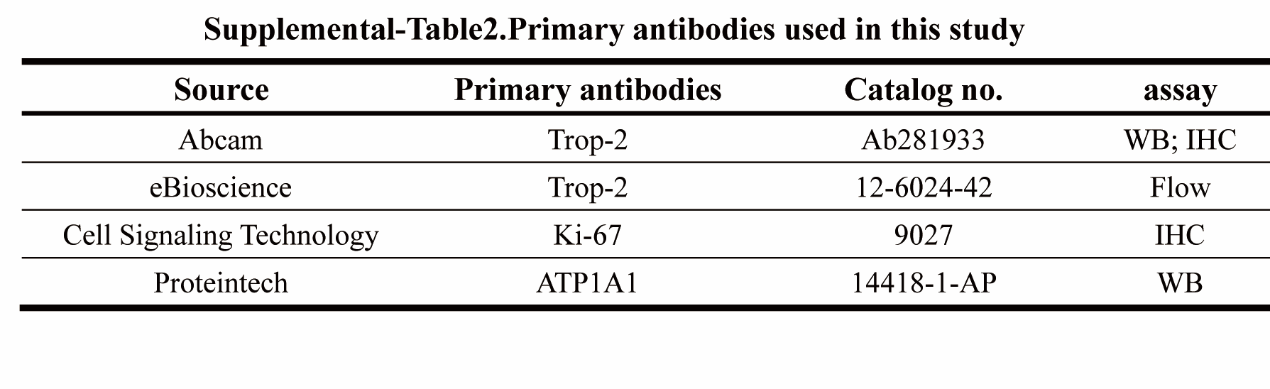

Supplement: Supplementary file 1 [file Supplementaryfile1.docx]
